# JC Virus T-Antigen Regulates Glucose Metabolic Pathways in Brain Tumor Cells

**DOI:** 10.1371/journal.pone.0035054

**Published:** 2012-04-09

**Authors:** Evan Noch, Ilker Kudret Sariyer, Jennifer Gordon, Kamel Khalili

**Affiliations:** Department of Neuroscience and Center for Neurovirology, Temple University School of Medicine, Philadelphia, Pennsylvania, United States of America; University of Michigan School of Medicine, United States of America

## Abstract

Recent studies have reported the detection of the human neurotropic virus, JCV, in a significant population of brain tumors, including medulloblastomas. Accordingly, expression of the JCV early protein, T-antigen, which has transforming activity in cell culture and in transgenic mice, results in the development of a broad range of tumors of neural crest and glial origin. Evidently, the association of T-antigen with a range of tumor-suppressor proteins, including p53 and pRb, and signaling molecules, such as β-catenin and IRS-1, plays a role in the oncogenic function of JCV T-antigen. We demonstrate that T-antigen expression is suppressed by glucose deprivation in medulloblastoma cells and in glioblastoma xenografts that both endogenously express T-antigen. Mechanistic studies indicate that glucose deprivation-mediated suppression of T-antigen is partly influenced by 5′-activated AMP kinase (AMPK), an important sensor of the AMP/ATP ratio in cells. In addition, glucose deprivation-induced cell cycle arrest in the G1 phase is blocked with AMPK inhibition, which also prevents T-antigen downregulation. Furthermore, T-antigen prevents G1 arrest and sustains cells in the G2 phase during glucose deprivation. On a functional level, T-antigen downregulation is partially dependent on reactive oxygen species (ROS) production during glucose deprivation, and T-antigen prevents ROS induction, loss of ATP production, and cytotoxicity induced by glucose deprivation. Additionally, we have found that T-antigen is downregulated by the glycolytic inhibitor, 2-deoxy-D-glucose (2-DG), and the pentose phosphate inhibitors, 6-aminonicotinamide and oxythiamine, and that T-antigen modulates expression of the glycolytic enzyme, hexokinase 2 (HK2), and the pentose phosphate enzyme, transaldolase-1 (TALDO1), indicating a potential link between T-antigen and metabolic regulation. These studies point to the possible involvement of JCV T-antigen in medulloblastoma proliferation and the metabolic phenotype and may enhance our understanding of the role of viral proteins in glycolytic tumor metabolism, thus providing useful targets for the treatment of virus-induced tumors.

## Introduction

JC virus (JCV) is the causative agent of the fatal human demyelinating disease, progressive multifocal leukoencephalopathy (PML), and has also been associated with multiple tumors of the central nervous system, including astrocytomas, glioblastomas, neuroblastomas, and medulloblastomas [1], [2] These CNS tumors can be marked by highly aggressive courses, with five-year survivals ranging from 50% in less aggressive forms to just 4% for patients with glioblastoma (Central Brain Tumor Registry of the United States, CBTRUS). Though there are many ongoing studies involved in the discovery of genetic factors underlying malignant tumorigenesis, especially pathways involved in cell survival and angiogenesis, there has been relatively limited research pertaining to the role of oncogenic viruses in the progression of solid tumors.

One of the key viral regulatory proteins of JCV, T-antigen, has been shown to be associated with human brain tumor formation. For example, JCV T-antigen protein expression can be detected by immunohistochemistry in as many as 50% of human brain tumors [1], [3]. Furthermore, JCV T-antigen-mediated transformation is known to occur in cells of neural origin, further implicating this oncogene in the pathogenesis of malignant brain tumors. On a molecular level, cells expressing T-antigen exhibit properties of immortalization, such as morphological changes, rapid doubling time, anchorage-independent growth, and production of flank tumors in nude mice [4]. Moreover, JCV T-antigen has been shown to deregulate cell cycle machinery through binding and inactivation of the tumor suppressors, p53 and pRb [5]–[7], and can augment expression of c-myc through β-catenin and LEF-1 [8]. Though these studies have provided useful insight into the transforming abilities of T-antigen, there have been few studies examining the regulation of endogenous T-antigen expression in brain tumors and the effect of tumoral physiological processes on this expression. In addition, there have not been any studies examining the effect of T-antigen on glycolysis or metabolic pathways utilized during tumor pathogenesis.

Glucose metabolism regulates the growth of many solid tumors, and the widely known observation that tumor cells exhibit much-enhanced glycolytic rates to satisfy the need for increased ATP demand, known as the Warburg effect [9], underlies much of a tumor's growth potential. Tumor cells also utilize glucose at an increased rate to maintain reducing equivalents of the reduced form of nicotinamide adenine dinucleotide (NADPH) and to limit the production of reactive oxygen species (ROS). Therefore, we investigated the effect of glucose deprivation on T-antigen expression and cell cycle regulatory and metabolic control mediated by T-antigen under these conditions. In this study, we have found that JCV T-antigen is downregulated under conditions of glucose deprivation in brain tumor-derived cell lines endogenously expressing JCV T-antigen and that T-antigen interacts with the 5′-adenosine monophosphate (AMP)-activated protein kinase (AMPK) pathway and exerts control over cell cycle and glucose metabolic pathways. These findings expand our current knowledge regarding mechanisms of T-antigen transformation and implicate this oncogene in metabolic pathways underlying tumorigenesis.

## Methods

### Cell Culture and Reagents

The human glioblastoma cell line, U-87MG, was obtained from ATCC. The mouse medulloblastoma cell lines expressing T-antigen, BsB8, as well as those not expressing T-antigen, Bs1a and Bs1f, were previously described [2]. HJC-2 cells were derived from glioblastomas generated in hamsters intracerebrally inoculated with JCV [10] and are tumorigenic *in vivo* [11]. All cell lines used were maintained in Dulbecco's modified Eagle's medium (DMEM) containing 10% fetal bovine serum and 100 units/mL penicillin and 100 ug/mL streptomycin. For experiments with glucose deprivation, DMEM containing 10% dialyzed FBS and either 1 g/L glucose or no glucose was utilized. All cells were plated at exactly the same cell number and were of similar confluence at the time of each experiment. Efforts were made to treat each well exactly the same during washes and medium changes to minimize intra-experimental variability. The construction of CMV-cyclin E was described previously [12]. N-acetylcysteine (Sigma) was used at a final concentration of 24 mM, and pyruvate (Gibco) was used at a final concentration of 1 mM. Fenofibrate (Sigma), 2-deoxy-D-glucose (Sigma), sodium oxamate (Sigma), 6-aminonicotinamide (Cayman Chemical), and oxythiamine (MP Biomedicals) were used at the indicated doses.

### Construction of Adenoviral vector capable of expressing JCV T-antigen

Cloning of an intronless JCV T-antigen [13] as a glutathione S-transferase (GST) fusion protein in the pGEX2T plasmid was described earlier [14]. Adenoviral vector construction was performed by Dr. Satish Deshmane, using a commercially available kit from Microbix Inc. (Ontario, Canada). JCV T-antigen DNA coding sequences were PCR-amplified, digested with EcoRI, and cloned into the EcoRI site of pDC515io, an adenoviral shuttle plasmid with an MCMV promoter. Recombinant clones were sequenced to confirm authenticity and proper orientation. This recombinant plasmid and another plasmid named pBHGfrt(del)E1,3FLP that provided an adenovirus type-5 genomic-backbone with E1 and E3 gene deletion were further used to co-transfect HEK 293 IQ cells. A recombinant adenovirus generated as a result of site-specific recombination was isolated, amplified, and further purified by cesium chloride density equilibrium banding. The construction of Ad-Null, a control adenovirus without any transgene, was described earlier [15].

### Construction of JCV large T-antigen shRNA

The shRNA construct for large T-antigen was described previously [16]. Briefly, shRNA constructs were generated to target the nucleotide sequences 139 to 162 of the JCV large T-antigen cDNA. PCR products carrying sense and antisense oligonucleotides of large T-antigen were cloned into the pLL3.7 vector. The viruses were packaged in 293T (human embryonic kidney) cells according to the procedure described previously. HJC-2 cells were seeded in 6-well plates at 50% confluency and were incubated with 1 mL of viral supernatants. The infected cells were then incubated in regular complete medium (1× DMEM, 10% FBS) for 48 hours. Inspection by fluorescence microscopy confirmed the presence of more than 80% of GFP-positive cells after viral infection.

### Cell Extraction and Western Blot Analysis

Whole-cell, cytoplasmic, nuclear protein extraction, and western blot were performed as previously described [17]. Antibodies used were as follows: SV40 Large T-antigen (Calbiochem, pAb416), Phospho-AMPK (Thr172) (Cell Signal), AMPK (Cell Signal), cyclin B1 (Santa Cruz), Cdc2 p34 (Santa Cruz), cyclin E (Calbiochem), cyclin A (Santa Cruz), G6PDH (Sigma), PKM2 (Cell Signal), TALDO1 (Cell Signal), and α-tubulin (Sigma). Immunoblots were developed using the appropriate secondary horseradish peroxidase-coupled antibodies and an enhanced chemiluminescence plus (ECL) kit (Pierce).

### ATP Measurement

ATP measurement was performed according to the manufacturer's instructions (Roche). Briefly, cells were lysed and were then incubated with a luciferase reagent, which converts D-luciferin, ATP, and oxygen to oxyluciferin, AMP, diphosphate, carbon dioxide, and light. Luciferase values were measured on a tube luminometer (Zylux Corporation), and values were plotted on an ATP standard curve to obtain the concentration of ATP within cells. All values were normalized to total protein levels in each sample, and each experiment was conducted in triplicate.

### Immunocytochemistry and Immunohistochemistry

For immunocytochemical studies, cells were plated in chamber slides and were then treated with glucose deprivation. Subsequently, cells were fixed in 4% formaldehyde in PBS for 15 minutes at room temperature. Cells were blocked in 5% normal goat serum for 2 hours at room temperature before staining with the appropriate primary antibodies overnight at 4°C. Cells were incubated with 1∶500 dilution of the appropriate secondary antibody and were then mounted with Vectashield mounting medium containing DAPI (Vector Laboratories). Cells were then analyzed by fluorescence microscopy on a Nikon Eclipse TE300 microscope with Slidebook 5 software (Intelligent Imaging Innovations).

For immunohistochemical analysis, brains were fixed in 4% buffered formalin, embedded in paraffin, and sectioned at 4 µm for histological and immunohistochemical analysis. Sections were deparaffinized in xylene and were rehydrated through successive incubations in decreasing concentrations of ethanol. Light microscopy was then performed on sections stained with hematoxylin and eosin (H&E). For immunohistochemical analysis, nonenzymatic antigen retrieval in citrate buffer was then performed for all antibodies by heating the slides to 95°C in 0.01 M citrate (pH 6.0) for 30 min. The sections were then rinsed in water followed by PBS and incubated in methanol containing 6% H_2_O_2_ for 20 minutes to quench endogenous peroxidase. Sections were then blocked using 5% normal goat serum and were incubated with the appropriate primary antibodies overnight at room temperature in a humidified chamber. Immunohistochemistry was then performed using the avidin–biotin–perioxidase complex system according to the manufacturer's instructions (Vectastain Elite ABC-Peroxidase Kit, Vector Laboratories). Images were acquired on an Olympus AX70 microscope with cellSens Entry software (Olympus).

### Cell Cycle Analysis

All detached or dead cells in the medium were collected, and remaining cells were then trypsinized and collected. The cell cycle distribution was measured using Guava Cell Cycle reagent (Guava Technologies). The percentage of cells in each phase of the cell cycle was calculated according to the manufacturer's instructions. Each experiment was conducted in triplicate.

### Reactive Oxygen Species (ROS) Detection

Cells were plated in chamber slides in either control media or glucose deprivation media as described above, washed, and then labeled with 25 µM carboxy-H_2_-DCFDA for 30 minutes at 37°C according to the manufacturer's instructions (Molecular Probes). In the presence of ROS, the reduced carboxy-DCFH is converted to carboxy-DCF and fluoresces green. Cells were counterstained with Hoechst 33342 dye during the last 5 minutes of incubation. Cells were then mounted in warm buffer and analyzed by fluorescence microscopy on a Nikon Eclipse TE300 microscope with Slidebook 5 software (Intelligent Imaging Innovations). ROS levels were calculated by measuring the pixel intensity in triplicate images using Adobe Photoshop CS software (Adobe).

### Measurement of Cell Viability

All detached or dead cells in medium were collected, and remaining cells were then trypsinized and collected. Cell viability was measured using Guava ViaCount reagent (Guava Technologies). The total number of cells as well as the total number of viable cells was counted. Each experiment was conducted in triplicate.

### Nude Mouse Tumor Studies

All experiments were conducted in accordance with the Temple University Institutional Care and Animal Use Committee (IACUC). For intracranial tumor implantation, one-million HJC-2 cells were implanted intracranially into the brains of nude mice at the following stereotaxic coordinates relative to bregma: −1.5 posterior, +2.5 lateral, and a depth of −3.5. Animals were weighed twice a week to monitor changes in weight and appetite. Three weeks after injection, animals were euthanized, and brains were sectioned at 400 µm in ice-cold sectioning buffer consisting of Gey's balanced salt solution, 15% D-glucose, 50 µg/mL gentamycin, and 50 µg/mL amphotericin-B. Following sectioning, slices were kept in ice-cold sectioning buffer for 30 minutes. Slices were then placed on microfilters (0.4 um Millicell-CM, Millipore, Inc., Bedford, MA) in 6-well plates in 1 mL/well of slice culture medium consisting of 25% OPTIMEM, 25% fetal bovine serum (FBS), 50% Hanks Balanced Salt Solution (HBSS), 50 µg/mL gentamycin, and 50 µg/mL amphotericin-B. After two days, slice cultures were treated with glucose deprivation or control medium for the indicated time points. Subsequently, tissue slices were snap-frozen for whole-cell extraction or processed for histological evaluation.

### Statistical Analysis

All experiments were conducted in triplicate, and results were analyzed using a two-tailed Student's t-test where indicated. Results with a p-value<0.05 were assigned statistical significance.

## Results

### JCV T-antigen is downregulated by glucose deprivation in T-antigen-expressing cells

**Figure 1:**
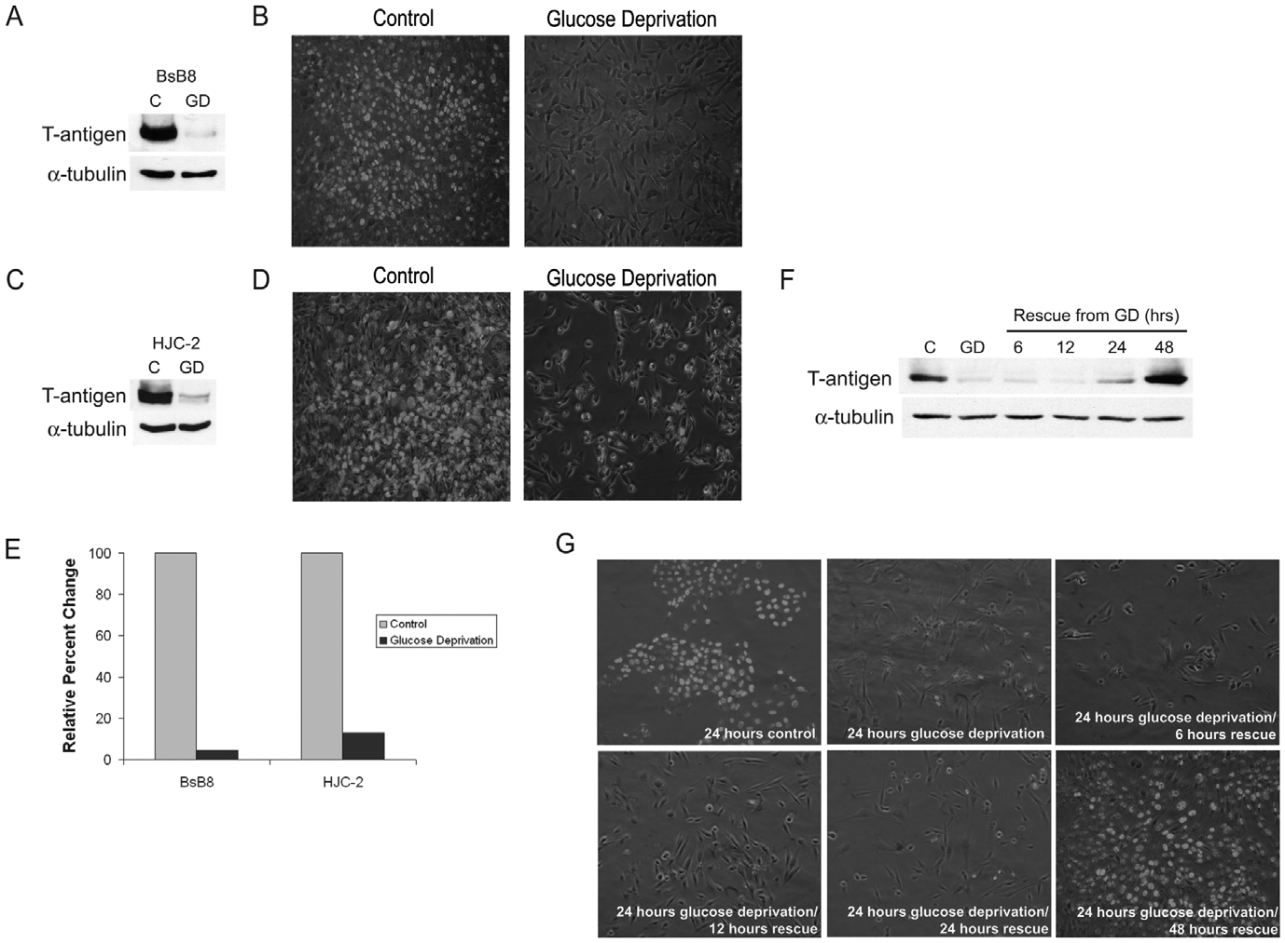
JCV T-antigen is downregulated by glucose deprivation in vitro. Endogenous JCV T-antigen expression was monitored in the cell lines, BsB8 (A and B) and HJC-2 (C and D), after 24-hour glucose deprivation by both western blot and immunocytochemical detection of T-antigen. E. Expression of T-antigen during conditions of glucose deprivation in the above cell lines was quantified. BsB8 cells were exposed to glucose deprivation for 24 hours and were then exposed to a subsequent time-course of normal medium containing 1 g/L D-glucose, after which T-antigen expression was measured by western blot (F) and immunocytochemistry (G). C, control; GD, glucose deprivation.

Since tumor cells utilize glucose at an increased rate and given findings of glucose metabolic regulation by SV40 small t-antigen [18] we investigated the effect of glucose deprivation on T-antigen expression. For these studies, we used the mouse medulloblastoma cell line, BsB8, which was isolated from medulloblastoma tumors induced in transgenic mice expressing the JCV early region [2]. Using both western blot and immunocytochemistry for T-antigen, we found that glucose deprivation significantly downregulated endogenous T-antigen protein expression in these cells after 24-hour treatment (Figure 1a, b, and e). We also observed decreased expression of small t-antigen during glucose deprivation (data not shown). We found similar results in HJC-2 cells, a cell line that was isolated from glioblastoma tissue induced in hamsters which were intracerebrally inoculated with JC virus (Figure 1c, d, and e). This cell line provides a unique benefit in that the virus itself without any prior genetic manipulation was able to transform cells and induce brain tumor formation, further lending support for the transformative properties of JCV. In BsB8 cells, this glucose deprivation-mediated T-antigen downregulation was able to be rescued by placing these cells into normal glucose-containing medium (1 g/L glucose) for an additional 24 or 48 hours (Figure 1f and g). Exposure to glucose deprivation for more than 48 hours led to some degree of cell death in BsB8 and HJC-2 cells (data not shown). In BsB8 cells, transcriptional studies using luciferase assays demonstrated an approximately 2-fold decrease in the activity of the JCV early promoter that controls T-antigen expression (data not shown). Therefore, it appears that the substantial decrease in T-antigen protein expression during glucose deprivation is a result of post-transcriptional or post-translational processing. These results indicate that T-antigen expression may be regulated by pathways involved in glucose metabolism under various stress conditions.

### AMPK activation mediates T-antigen downregulation, and T-antigen suppresses AMPK activation during glucose deprivation

**Figure 2:**
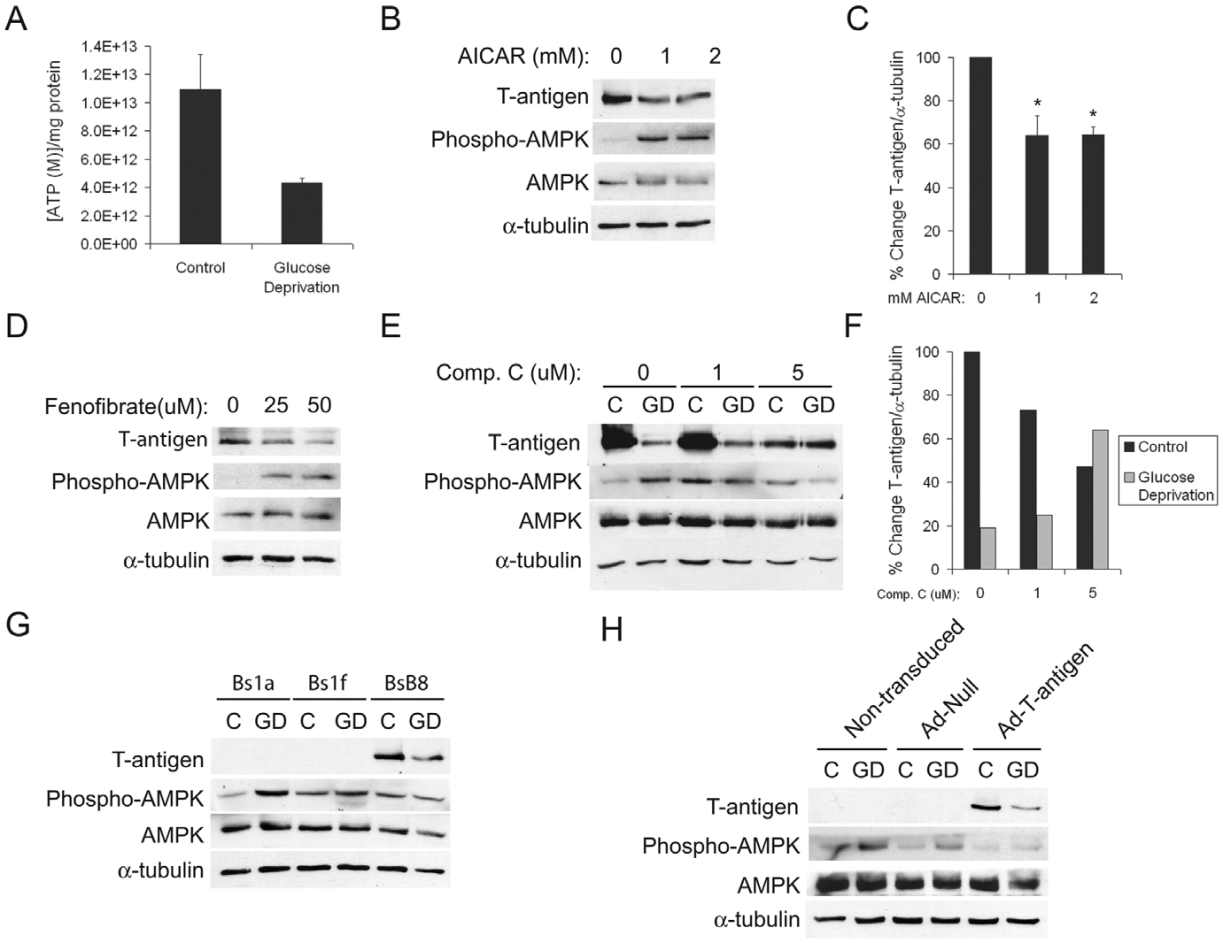
T-antigen and AMPK exhibit reciprocal repression during glucose deprivation. A. BsB8 cells were treated with glucose deprivation for 24 hours, and ATP levels were quantified. Results are presented as the concentration of ATP and are normalized to the total protein content of each sample. The experiment was conducted in triplicate. B. BsB8 cells were treated with the indicated doses of the AMPK activating agent, AICAR, for 24 hours, and T-antigen expression was measured by western blot. C. Quantification of T-antigen expression in B. (*, p<0.05). D. BsB8 cells were treated with fenofibrate at the indicated doses for 24 hours, and T-antigen and phosphorylated AMPK levels were determined by western blot. E. BsB8 cells were treated with various doses of the inhibitor of AMPK activation, Compound C, or DMSO, under conditions of glucose deprivation for 24 hours, and T-antigen expression was measured by western blot. F. Quantification of T-antigen expression in E. G. Bs1a, Bs1f, and BsB8 cells were exposed to glucose deprivation for 24 hours, and T-antigen expression and AMPK phosphorylation were measured by western blot. H. U-87MG cells that were transduced with adenovirus expressing T-antigen, Null, or were non-transduced were exposed to glucose deprivation for 24 hours, and T-antigen and phosphorylated AMPK levels were measured by western blot. C, control; GD, glucose deprivation.

Since it is well known that glucose deprivation leads to an increase in the [AMP]/[ATP] ratio and subsequent activation of AMP-activated protein kinase (AMPK) [19], we investigated the involvement of this pathway in glucose deprivation-mediated T-antigen downregulation. We first demonstrated that BsB8 cells exhibit a decreased level of ATP during glucose deprivation (Figure 2a). We found that treatment of BsB8 cells with AICAR, an activator of AMPK, resulted in significant T-antigen downregulation (Figure 2b and c). Additionally, treatment of these cells with fenofibrate, a peroxisome proliferator-activated receptor (PPAR)α agonist that also activates the AMPK pathway, resulted in T-antigen downregulation (Figure 2d). Furthermore, we demonstrated that treatment with a specific AMPK inhibitor, Compound C [20], resulted in rescue of T-antigen expression under glucose deprivation conditions (Figure 2e and f). These data demonstrate that AMPK activation is required for T-antigen downregulation during periods of low glucose and that activation of AMPK is sufficient to decrease T-antigen expression. Since it has been shown that SV40 T-antigen can induce AMPK activation during periods of glucose starvation [18], we also wanted to investigate the role of JCV T-antigen in glucose deprivation-induced AMPK activation. For these studies, we used BsB8 cells as well as the clones, Bs1a and Bs1f, which were derived from the same original medulloblastoma tumor tissue but which do not express T-antigen [2]. Though Bs1a and Bs1f cells are not identical to BsB8 cells with respect to the extent of their tumorigenic activity, they provide a useful model to study the impact of T-antigen expression on the metabolic phenotype as well as potential reasons for the lack of T-antigen expression in these cells. We found less phosphorylation of AMPK in BsB8 cells as compared to Bs1a or Bs1f cells, indicating that T-antigen can suppress activation of AMPK under periods of glucose deprivation (Figure 2g). Alternatively, the time necessary to observe significant AMPK phosphorylation may be prolonged in BsB8 relative to Bs1a or Bs1f cells, indicating initial differential responses to cell stress in these cell types. In order to determine whether T-antigen protein can be downregulated by glucose deprivation in other cell types, we confirmed decreased expression of T-antigen during glucose deprivation by transducing U-87MG cells with an adenovirus expressing either T-antigen or a null construct. Similarly to cells endogenously expressing T-antigen, U-87MG cells expressing T-antigen exhibited less AMPK phosphorylation during glucose deprivation than null- or non-transduced controls (Figure 2h). Though T-antigen may not be required for transformation of these cells, T-antigen does clearly influence the metabolic response to glucose deprivation in these cells. Together, these findings indicate a potential negative feedback loop between JCV T-antigen and AMPK activation during glucose deprivation, a pathway that may regulate metabolic utilization of glucose in T-antigen-positive tumors.

### AMPK activation induces G1 and G2 cell cycle arrest and reduced cyclin expression, which modulates cell cycle phase-regulated T-antigen expression

**Figure 3:**
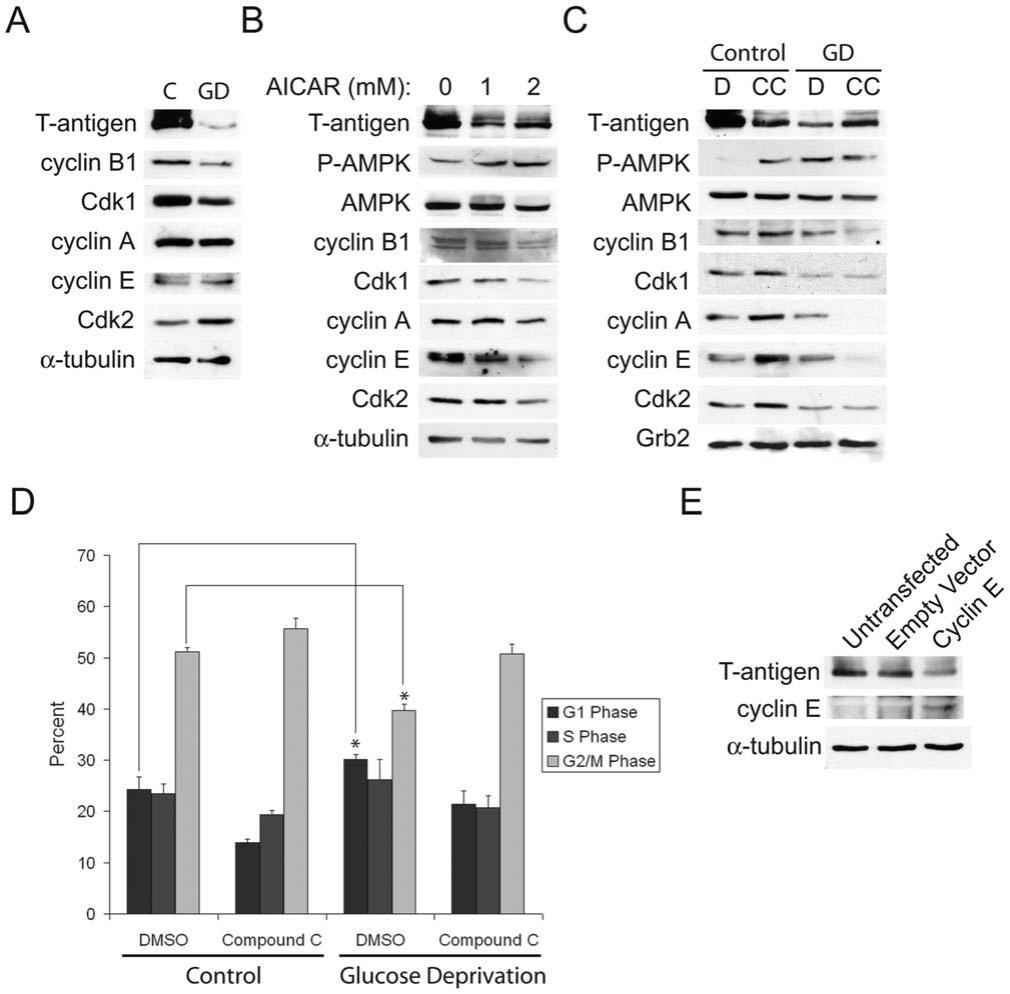
T-antigen downregulation during glucose deprivation is associated with AMPK-mediated cell cycle control. A. BsB8 cells were exposed to glucose deprivation for 24 hours, and the expression of T-antigen and cell cycle regulators was monitored by western blot. B. BsB8 cells were exposed to the indicated doses of AICAR, and the expression of T-antigen and cell cycle regulators was measured by western blot. C. BsB8 cells were treated with 5 uM Compound C (CC) or DMSO (D) and exposed to glucose deprivation for 24 hours, and the expression of cell cycle regulators was monitored by western blot. D. Cell cycle analysis using Guava cell cycle reagent was performed in cells from C. (*, p<0.05). E. BsB8 cells were transfected with CMV-cyclin E or empty vector, and T-antigen expression was monitored by western blot. C, control; GD, glucose deprivation.

The link between AMPK activation and cell cycle regulation has been investigated in many studies involving tumor pathogenesis [21], [22]. Specifically, it has been demonstrated that AMPK activation results in decreased levels of cyclin A, cyclin B1 and cyclin E, but these changes may depend on the particular method of activation [23], [24]. During glucose deprivation, we found that BsB8 cells exhibit downregulation of cyclin B1 and Cdk1 but no significant changes in the expression of cyclin A (Figure 3a). AMPK activation using AICAR alone was able to mimic the effects of glucose deprivation on cyclin regulation as well, with similar downregulation of cyclin B1 and Cdk1 (Figure 3b). Interestingly, AMPK inhibition during glucose deprivation using Compound C, which prevented T-antigen downregulation, also prevented decreased cyclin expression (Figure 3c). This cyclin regulation also led to concomitant alterations in cell cycle phase control. Whereas glucose-deprived cells exhibited arrest in the G1 phase and less G2 accumulation, AMPK inhibition prevented G1 arrest and caused increased G2 accumulation, which was associated with T-antigen rescue (Figure 3d). Previous studies have noted that polyomaviruses tend to preferentially arrest cells in the G2 phase of the cell cycle in order to replicate [25], so AMPK inhibition-mediated T-antigen rescue during glucose deprivation would enhance T-antigen-induced proliferation under cell stress conditions in the tumor microenvironment. BsB8 cells transfected with cyclin E, which acts as an inducer of G1 arrest, also caused T-antigen downregulation, indicating that conditions that favor G1 arrest result in reduction in T-antigen expression (Figure 3e).

### T-antigen induces expression of cyclin B1 and leads to enhanced G2 accumulation

**Figure 4:**
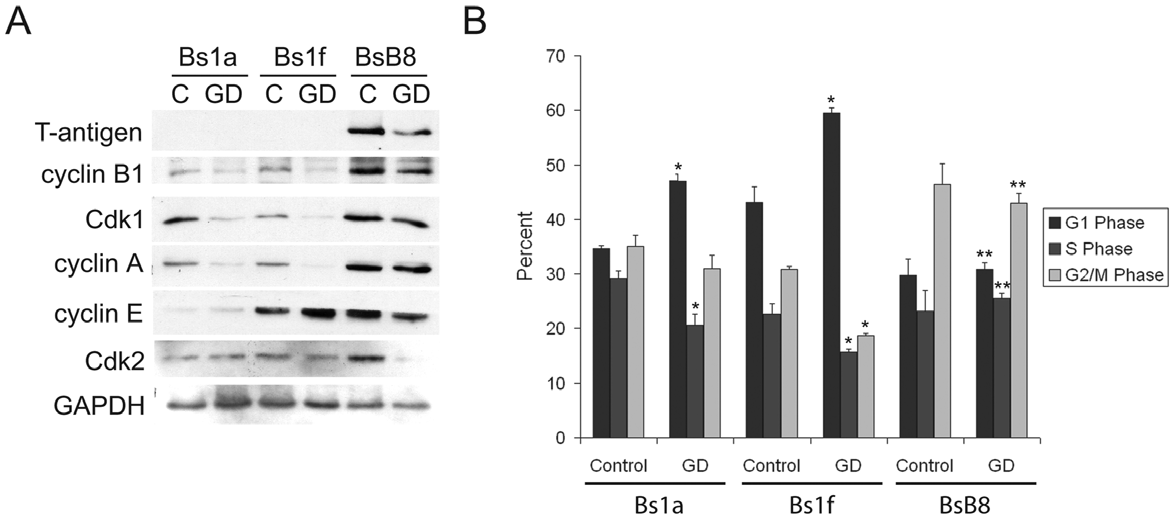
T-antigen prevents G1 arrest and preferentially arrests cells in the G2 phase in glucose-deprived cells. A. Bs1a, Bs1f, and BsB8 cells were treated with glucose deprivation or control medium for 16 hours and were subsequently processed for whole-cell protein extraction for western blot analysis of cell cycle regulatory proteins. B. Identical samples from A. were processed for cell cycle analysis using Guava cell cycle reagent. (* = control compared to GD, p<0.05; # = BsB8 compared to Bs1a and Bs1f cells, p<0.05). C, control; GD, glucose deprivation.

Because T-antigen is downregulated by glucose deprivation, which has been shown to induce G1 arrest, we sought to determine the effect of T-antigen on cyclin expression and the functional consequences on cell cycle distribution. For these studies, we used the T-antigen-expressing BsB8 cells and the T-antigen non-expressing cells, Bs1a and Bs1f, described above. When we compared the cell cycle expression profile of these cells under glucose deprivation, we found that BsB8 cells expressed higher levels of cyclin B1 and cyclin A relative to Bs1a and Bs1f cells (Figure 4a). Furthermore, when we compared the cell cycle distribution of these cells under glucose deprivation, we found that BsB8 cells were significantly more resistant to G1 arrest and exhibited significantly higher percentages of cells in the G2 phase than Bs1a and Bs1f cells (Figure 4b). These findings indicate that T-antigen maintains cells in the G2 phase of the cell cycle, even in the midst of G1-arresting cell stress signals.

### T-antigen reduces ROS accumulation, prevents decreased ATP levels, and prevents cytotoxicity during glucose deprivation

**Figure 5:**
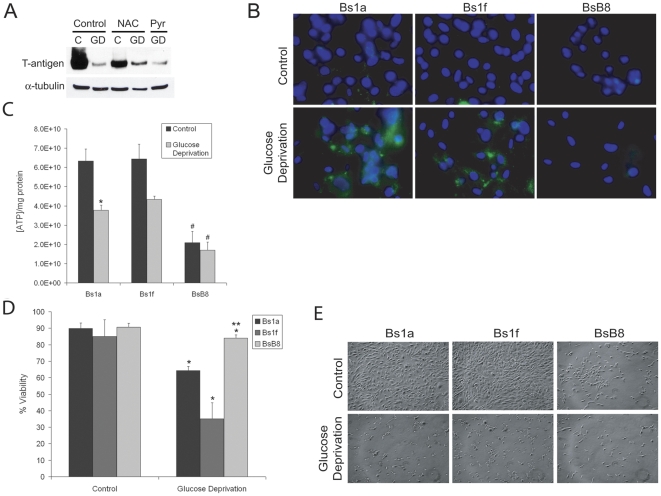
T-antigen prevents glucose deprivation-induced ROS production, reduction in ATP levels, and cytotoxicity. A. BsB8 cells were incubated with 24 mM N-acetylcysteine (NAC), 1 mM pyruvate, or were untreated and then exposed to glucose deprivation or control medium for 24 hours. Pyruvate was dissolved in a small volume of water and a negligible volume was added to the medium in each well. The expression of T-antigen was assessed in whole-cell extracts. B. Bs1a, Bs1f, or BsB8 cells were exposed to glucose deprivation or control medium for 16 hours, and ROS production was measured using the fluorescent dye, 25 µM carboxy-H_2_-DCFDA. Hoechst staining was also performed to label nuclei. C. ROS levels were quantified by calculating the mean pixel intensity in triplicate images acquired. (* = control compared to GD, p<0.05; # = BsB8 compared to Bs1f, p<0.05). D. Bs1a, Bs1f, or BsB8 cells were exposed to glucose deprivation for 16 hours, and ATP levels were then measured. The levels of ATP per cell were measured and were normalized to the total protein present in each sample. E. Bs1a, Bs1f, or BsB8 cells were exposed to glucose deprivation or control medium for 24 hours, and cell viability was measured using Guava ViaCount reagent. (* = control compared to GD, p<0.05; # = BsB8 compared to Bs1a and Bs1f cells, p<0.05). F. Phase-contrast images of cells treated with glucose deprivation in D. C, control; GD, glucose deprivation.

It is well known that glucose deprivation induces production of reactive oxygen species (ROS) through decreased production of NADPH, the reduced form of nicotinamide adenine dinucleotide phosphate (NADP^+^), and a loss of reductive potential in the cell [26]. Therefore, we hypothesized that ROS production during glucose deprivation may play a role in T-antigen downregulation. Treatment of BsB8 cells with the thiol antioxidant, N-acetylcysteine (NAC), completely prevented T-antigen downregulation from glucose deprivation (Figure 5a). However, treatment of these cells with pyruvate, a weaker anti-oxidant than NAC which has also been shown to act as an intracellular scavenger of ROS [27], did not prevent T-antigen downregulation. These data demonstrate that the induction of ROS during glucose deprivation may be important for the observed decrease in T-antigen expression.

To examine the importance of T-antigen expression on ROS production during glucose deprivation, we used BsB8 cells as well as the T-antigen non-expressing clones, Bs1a and Bs1f. We found that ROS production was significantly reduced during glucose deprivation in BsB8 cells as compared to Bs1f cells (Figure 5b). BsB8 cells did not exhibit any increase in ROS production during glucose deprivation, and there was a general increase in ROS production in Bs1a cells during glucose deprivation with the difference between these cells and BsB8 cells in this condition approaching statistical significance (p = 0.05). These findings suggest that T-antigen is able to prevent ROS accumulation that would lead to subsequent cytotoxicity during glucose deprivation. During glucose deprivation, cells produce decreased ATP as a result of a reduction in glycolytic rates and subsequent decline in intermediates to be used in the mitochondrial electron transport chain. Therefore, we investigated whether T-antigen affects the production of ATP during periods of glucose starvation. As compared to Bs1a and Bs1f cells, which demonstrated a reduction in ATP levels under conditions of low glucose, BsB8 cells were more resistant to diminished ATP production (Figure 5d). Furthermore, BsB8 cells produced significantly less ATP under control conditions, indicating that these cells may demonstrate a different metabolic phenotype, may be less dependent on ATP production, and may utilize cellular energy stores in alternative manners.

We next wanted to investigate the functional significance of glucose deprivation on survival of cells expressing endogenous T-antigen. Since glucose deprivation induces rapid cytotoxicity because of the resulting lack of reductive capacity within the cell, we investigated the impact of T-antigen expression on glucose deprivation-induced cell death. We found that BsB8 cells exhibited significantly less glucose deprivation-mediated cytotoxicity than Bs1a or Bs1f cells (Figure 5e and f). BsB8 cells can survive under these periods for a much longer amount of time than their T-antigen non-expressing counterparts. BsB8 cells survive past the 48-hour timepoint under conditions of glucose deprivation (data not shown), whereas this condition is uniformly lethal to the Bs1a and Bs1f cell lines. These results suggest that the presence of T-antigen during glucose deprivation may be protective against rapid cell death, potentially because of T-antigen-mediated alterations in energy metabolism and ROS production under periods of cell stress.

### T-antigen is downregulated through specific glycolytic pathways during glucose deprivation and regulates the glycolytic enzyme expression profile in medulloblastoma cells

**Figure 6:**
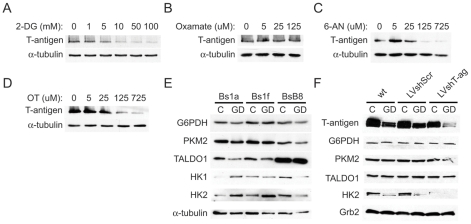
T-antigen is downregulated by glucose deprivation through specific glycolytic pathways and influences the glycolytic enzyme expression profile of medulloblastoma cells. BsB8 cells were treated with the indicated doses of 2-deoxy-D-glucose (2-DG) (A), oxamate (B), 6-aminonicotinamide (6-AN) (C), and oxythiamine (D) for 24 hours, and T-antigen expression was measured by western blot. E. Bs1a, Bs1f, and BsB8 cells were exposed to glucose deprivation for 24 hours, and glycolytic enzyme expression was measured by western blot. F. HJC-2 cells were transduced with lentivirus expressing Large T-antigen, scrambled shRNA, or were non-transduced and then exposed to glucose deprivation for 16 hours. Glycolytic enzyme expression was measured by western blot. G. Quantification of HEK2 expression relative to Grb2 in F. C, control; GD, glucose deprivation.

Given our findings of T-antigen downregulation by glucose deprivation and functional implications for cell survival, we next studied the regulation of T-antigen at the glycolytic level. For these studies, we used inhibitors of several key glycolytic enzymes to determine the role of glycolysis in the regulation of endogenous T-antigen expression. Treatment of BsB8 cells with 2-deoxy-D-glucose (2-DG), which inhibits glycolysis after initial phosphorylation by hexokinase, induced T-antigen downregulation in a dose-dependent manner (Figure 6a). To further identify glycolytic regulation of T-antigen, we used oxamate, an inhibitor of the enzyme, lactate dehydrogenase. We did not observe any appreciable changes in T-antigen expression at varying doses of oxamate, indicating that T-antigen regulation likely occurs upstream of lactate production (Figure 6b). In light of our findings demonstrating T-antigen prevention of ROS production during glucose deprivation, a process ameliorated by NADPH production from pentose phosphate pathway activity, we used inhibitors of this pathway to determine the T-antigen expression status with pentose phosphate pathway inhibition. Treatment of BsB8 cells with 6-aminonicotinamide (6-AN), an inhibitor of glucose 6-phosphate dehydrogenase (G6PDH), the rate-limiting enzyme in the pentose phosphate pathway, resulted in marked downregulation of T-antigen in a dose-dependent manner (Figure 6c). Similarly, inhibition of the transketolase enzyme, which catalyzes the production of fructose 6-phosphate and glyceraldehyde 3-phosphate from pentose phosphate intermediates, with oxythiamine resulted in T-antigen downregulation in a dose-dependent manner (Figure 6d). Therefore, it appears that T-antigen expression may be controlled through various metabolic pathways that regulate glucose breakdown.

In order to investigate whether T-antigen affects glycolytic metabolism in medulloblastoma cells, we compared the expression of glycolytic enzymes in the T-antigen-expressing BsB8 cells to the T-antigen non-expressing Bs1a and Bs1f cells during glucose deprivation. While we found no changes in the expression of G6PDH or the M2 isoform of pyruvate kinase (PKM2), which has been implicated in tumor metabolism and proliferation [28], we found significantly higher levels of transaldolase-1 (TALDO1) expression in BsB8 cells as compared to Bs1a and Bs1f cells, under control conditions as well as during glucose deprivation (Figure 6e). In addition, we found that BsB8 cells exhibit increased expression of hexokinase-2 (HK2) in control medium but do not exhibit HK2 upregulation, as occurs in Bs1a and Bs1f cells, during glucose deprivation. We confirmed these results using the glioblastoma cell line, HJC-2, and performed experiments using lentivirus expressing T-antigen shRNA, which has been shown previously to be effective at reducing T-antigen expression in this cell line by greater than 50% and has been shown to induce apoptosis at 72 hours post-transfection [16]. We transduced these cells with lentivirus expressing large T-antigen shRNA or scrambled shRNA or used wild-type cells. We then exposed these cells to 16 hours of glucose deprivation before measuring expression levels of glycolytic enzymes by western blot. In this paradigm, we found that cells expressing T-antigen shRNA exhibited downregulation of HK2 in control medium, verifying that T-antigen positively regulates HK2 expression (Figure 6f). We did not observe a significant decrease in PKM2 levels in cells treated with T-antigen shRNA, similar to results obtained in BsB8 cells. The difference between the HJC-2 and BSB8 cells in regards to their levels of TALDO1 may actually reflect lower endogenous levels in the T-antigen negative clones, rather than higher TALDO1 levels in the BSB8 cells. However, the overall trend in TALDO1 was similar in the HJC-2 versus the BSB8 cells. Taken together, these results suggest that T-antigen-mediated alterations in the expression of glycolytic enzymes may lead to subsequent modulation of glycolytic flux within tumor cells.

### T-antigen is downregulated by glucose deprivation in ex vivo tumor slice cultures

**Figure 7:**
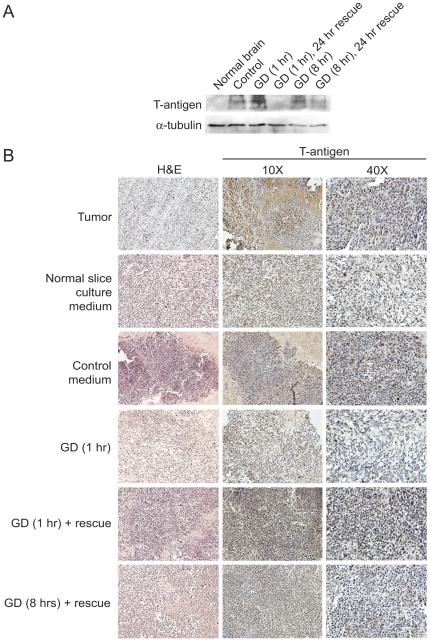
JCV T-antigen is downregulated by glucose deprivation in *ex vivo* glioblastoma xenograft slice cultures. A. HJC-2 brain xenografts were sectioned and cultured *in vitro* and were then treated with control medium or glucose deprivation, with and without rescue from glucose deprivation by subsequent incubation in control medium, or were untreated for various time-points and were harvested for whole tissue lysates. Subsequently, western blot analysis for T-antigen expression was performed. B. Hematoxylin and eosin (H&E) staining as well as immunohistochemical analysis for T-antigen expression were performed in tissue samples obtained from the xenograft slice cultures described in A. GD, glucose deprivation.

JCV T-antigen is a potent oncogenic protein that can interact with a variety of cellular proteins to promote tumorigenesis. In light of our findings of T-antigen downregulation by glucose deprivation, we examined the metabolic regulation of T-antigen in a semi-*in vivo* system. For these studies, we used an *ex vivo* tumor slice culture model with HJC-2 glioblastoma xenografts implanted into the brains of nude mice. Following tumor growth, brain slices containing tumor were prepared and were then grown *in vitro*. Tumor slice cultures were then exposed to various time-courses of glucose deprivation or rescue in normal medium before harvesting for western blot or immunohistochemical analysis. To reinforce our previous findings, we found that T-antigen is downregulated by 1 hour and 8 hours of glucose deprivation only appreciably after 24 hours of rescue in normal medium (1 g/L D-glucose) (Figure 7a). We were also able to identify reduction in T-antigen expression in HJC-2 tumor tissue exposed to 1 hour and 8 hours glucose deprivation plus rescue (Figure 7b). The changing pattern of T-antigen expression during the time course of GD and rescue paradigm suggests that mechanisms controlling the regulation of T-antigen expression may have alternative long-term and short-term effects and understanding these effects will require further study. These data indicate that T-antigen is also downregulated by glucose deprivation within tumor tissue and may provide useful information to develop strategies against T-antigen-positive brain tumors.

## Discussion

**Figure 8:**
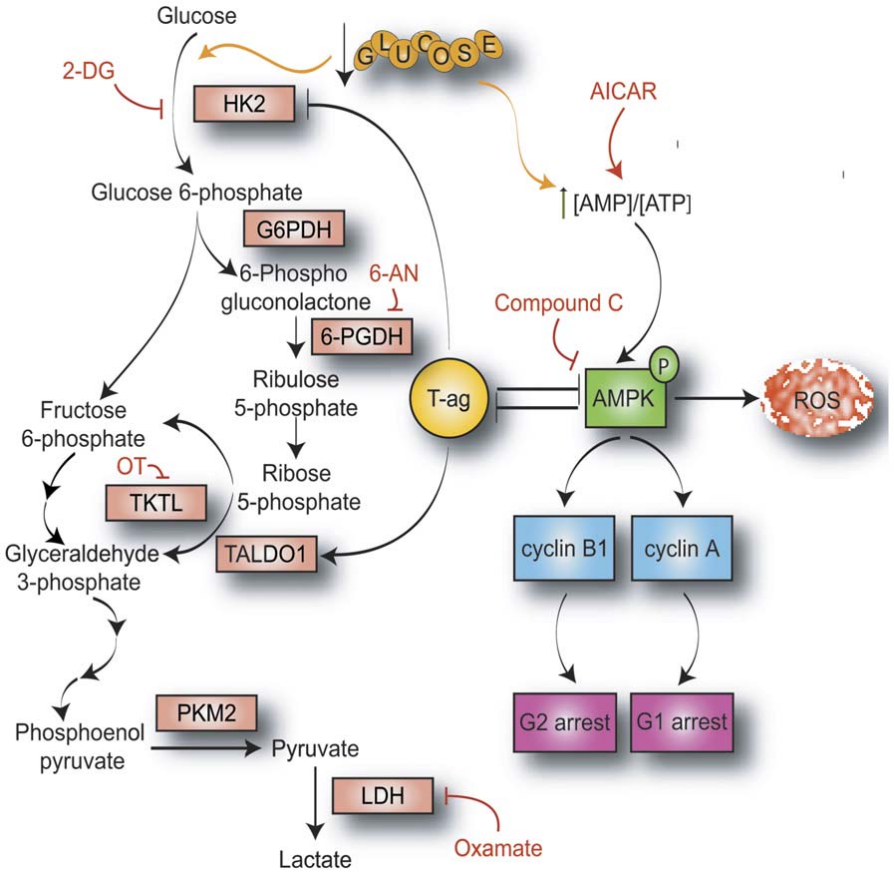
Mechanism and significance of metabolic signaling pathways affected by the presence of JCV T-antigen. JCV T-antigen is downregulated by glucose deprivation in an AMPK-dependent manner. During periods of glucose deprivation, T-antigen inhibits AMPK phosphorylation, which prevents the induction of reactive oxygen species (ROS) and subsequent cytotoxicity. Additionally, T-antigen relieves AMPK-mediated cyclin B1 and cyclin A inhibition, leading to decreased G1 arrest. Glucose deprivation induces both enhanced glycolytic flux to maintain high levels of ATP production as well as enhanced pentose phosphate pathway activation to supply reducing equivalents in the form of reduced nicotinamide adenine dinucleotide phosphate (NADPH) to counteract ROS production produced by glycolysis. T-antigen upregulates transaldolase-1 (TALDO1) expression to shift intermediates from the pentose phosphate pathway towards glycolysis to enhance ATP production and also prevents hexokinase 2 (HK2) upregulation during glucose deprivation. (HK2, hexokinase; G6PDH, glucose 6-phosphate dehydrogenase; 6-PGDH, 6-phoshogluconate dehydrogenase; TKTL, transketolase; PKM2, pyruvate kinase M2; LDH, lactate dehydrogenase; 2-DG, 2-deoxyglucose; 6-AN, 6-aminonicotinamide; OT, oxythiamine).

JC Virus (JCV) has the ability to induce multiple types of brain tumors in experimental animal models. On a molecular level, JCV can bind to and inactivate β-catenin as well as regulate key modulators of oncogenesis, including the IGF-IR and p53 pathways [6], [29], [30]. In this study, we have shown that T-antigen is downregulated by glucose deprivation in multiple types of JCV-transformed cell lines and that this downregulation is a product of glucose deprivation-mediated AMPK activation (Figure 8). Since JCV T-antigen may interact with multiple members of the AMPK pathway, it is likely that signaling through this pathway may alter the stability of T-antigen protein, leading to subsequent degradation. Given findings of AMPK-activated cell cycle arrest in the G1 phase of the cell cycle, our data support the possibility that T-antigen expression status may be linked to the phase of the cell cycle. Our data indicate that T-antigen-expressing BsB8 cells preferentially accumulate in the G2 phase of the cell cycle and that deviation from this cell cycle phase, which occurs through the process of glucose deprivation, induces T-antigen downregulation. Interestingly, this downregulation is lost if AMPK activation is inhibited, which causes G2 re-accumulation during glucose deprivation, further connecting T-antigen expression to the G2 phase. These findings are supported by publications demonstrating that several viruses preferentially induce G2 cell cycle arrest, such as HIV [31], HBV [32], and HPV [33]. Our results from studying the AMPK pathway are reinforced by the observation that T-antigen-expressing BsB8 cells exhibit significantly greater cyclin B1 expression and a greater percentage of cells in the G2 phase than the similar T-antigen non-expressing cells. In the context of glucose deprivation, T-antigen preferential arrest in the G2 phase may help to prevent G1 arrest and G1-mediated apoptosis, processes that can occur with a multitude of other cell stressors, such as UV exposure, hypoxia, and various chemotherapeutic agents. Therefore, cells expressing T-antigen may resist cytotoxic processes that are activated during the G1 phase.

Though T-antigen has previously been shown to induce tumor cell growth through regulation of multiple tumor suppressor pathways, these studies implicate T-antigen in the metabolic regulation of medulloblastoma cells. We observed that glucose deprivation reduced ATP levels in all cells tested, but BsB8 cells exhibited lower basal levels of ATP and less change in ATP levels during glucose starvation than Bs1a or Bs1f cells. Therefore, it appears that BsB8 cells produce less ATP under normal conditions, perhaps due to other pathways that fuel the metabolic needs of these cells. Alternatively, BsB8 cells may have evolved ways to develop more efficient pathways of energy utilization so that periods of starvation do not as greatly affect their growth as T-antigen non-expressing cells. Enhanced metabolic efficiency may also help to reduce the production of ROS, which do not accumulate to as great an extent in BsB8 as Bs1a and Bs1f cells. Our findings of reduced hexokinase 2 expression levels in BsB8 cells relative to Bs1a and Bs1f cells during glucose deprivation indicate that BsB8 cells may therefore import less glucose under starvation conditions but may actually upregulate this pathway under control conditions, as evidenced by results in HJC-2 cells. In this sense, BsB8 cells may be less glucose-dependent under conditions of cell stress than their T-antigen non-expressing counterparts, which undergo more rapid cell death when their critical energy source is depleted.

Given our observations that T-antigen-expressing cells exhibit increased expression of transaldolase-1 (TALDO1) but similar levels of glucose 6-phosphate dehydrogenase (G6PDH) and the M2 isoform of pyruvate kinase (PKM2), T-antigen may simultaneously support both glycolysis and the pentose phosphate pathway. This scenario has been shown for SV40-transformed cells, which demonstrate redistribution of membrane glucose transporters [34], increases in aerobic glycolysis [35], and increases in the activity of the transaldolase enzyme [36], indicating that SV40 is capable of regulating glycolytic precursors and the redox status maintained by pentose phosphate NADPH production. In one instance, enhanced TALDO1 expression may allow the maintenance of elevated ATP production to fulfill the needs of cells undergoing metabolic stress. On the other hand, enhanced TALDO1 expression could also rapidly deplete pentose phosphate intermediates and NADPH, thereby shifting more glucose 6-phosphate towards this pathway to sustain NADPH for reducing equivalents. The net effect of T-antigen would then be the acceleration of ATP production with concomitant maintenance of NADPH levels to counteract resultant ROS production, a complement of features that would greatly enhance the malignant potential of tumor cells.

The regulation of T-antigen expression by glycolytic inhibition also highlights the significant impact of viral oncoproteins on glycolytic metabolism. Many oncogenic viruses, including hepatitis C virus (HCV), human T-lymphotropic virus type 1 (HTLV-1), and human papilloma virus (HPV), have been implicated in alterations in glucose transport and glycolytic enzyme expression and activity [37]–[39]. In addition, similar results with glycolytic inhibition have been demonstrated with HPV E6/E7 proteins, whose transcription is blocked with the use of 2-DG, indicating that flux through this pathway may be responsible for the expression of HPV-targeted transcription factors [40] Furthermore, since 2-DG induces a reduction in viral-cell and cell-cell fusion [41], [42], this compound may also be protective against JCV infection, reactivation, and potentially JCV-associated malignancies. 2-DG has also been indicated for the treatment of glioblastoma [43] and other advanced solid malignancies and synergizes with compounds used to treat virus-derived cancers, such as carboplatin and paclitaxel [44]–[46]. Therefore, 2-DG in combination with currently approved chemotherapeutic regimens may be a useful therapeutic compound for the treatment of JCV-associated brain tumors.

Regulation of glucose metabolism continues to represent one of the most significant alterations in tumor cells as compared to normal cells. By increasing production of ATP and at the same time maintaining NADPH equivalents, tumor cells have evolved pathways to rapidly proliferate while avoiding many of the negative consequences of such uninhibited growth. In this study, we show that JC Virus T-antigen is downregulated by glucose deprivation through AMPK-dependent pathways, which also regulates cell cycle control under these conditions. Additionally, we indicate a new potential role for T-antigen in the regulation of the metabolic profile of tumor cells, which may have implications for human brain tumors that exhibit T-antigen expression. By better understanding the mechanism of T-antigen-mediated glycolytic regulation, it may be possible to develop novel therapeutics that would target T-antigen in human tumors, which could potentially influence both initial transforming events as well as continued growth in the midst of metabolic stress signals within the tumor microenvironment.

## References

[1] Del Valle L, Gordon J, Assimakopoulou M, Enam S, Geddes JF (2001). Detection of JC virus DNA sequences and expression of the viral regulatory protein T-antigen in tumors of the central nervous system.. Cancer Res.

[2] Krynska B, Otte J, Franks R, Khalili K, Croul S (1999). Human ubiquitous JCV(CY) T-antigen gene induces brain tumors in experimental animals.. Oncogene.

[3] Caldarelli-Stefano R, Boldorini R, Monga G, Meraviglia E, Zorini EO (2000). JC virus in human glial-derived tumors.. Human Pathol.

[4] Khalili K, Del Valle L, Otte J, Weaver M, Gordon J (2003a). Human neurotropic polyomavirus, JCV, and its role in carcinogenesis.. Oncogene.

[5] Caracciolo V, Macaluso M, D'Agostino L, Montanari M, Scheff J (2010). Cross-talk between T-Ag presence and pRb family and p53/p73 signaling in mouse and human medulloblastoma.. J Cell Biochem.

[6] Krynska B, Gordon J, Otte J, Franks R, Knobler R (1997). Role of cell cycle regulators in tumor formation in transgenic mice expressing the human neurotropic virus, JCV, early protein.. J Cell Biochem.

[7] Staib C, Pesch J, Gerwig R, Gerber JK, Brehm U (1996). p53 inhibits JC virus DNA replication in vivo and interacts with JC virus large T-antigen.. Virology.

[8] Gan DD, Reiss K, Carrill T, Del Valle L, Croul S (2001). Involvement of Wnt signaling pathway in murine medulloblastoma induced by human neurotropic JC virus.. Oncogene.

[9] Warburg O (1956). On the origin of cancer cells.. Science.

[10] Wold WS, Green M, Mackey JK, Martin JD, Padgett BL (1980). Integration pattern of human JC virus sequences in two clones of a cell line established from a JC virus-induced hamster brain tumor.. J Virol.

[11] Raj G, Gordon J, Logan T, Hall D, Deluca A (1995). Characterization of glioma-cells derived from human polyomavirus-induced brain-tumors in hamsters.. Int J Oncol.

[12] Hinds PW, Mittnacht S, Dulic V, Arnold A, Reed SI (1992). Regulation of retinoblastoma protein functions by ectopic expression of human cyclins.. Cell.

[13] Bollag B, Mackeen PC, Frisque RJ (1996). Purified JC virus T antigen derived from insect cells preferentially interacts with binding site II of the viral core origin under replication conditions.. Virology.

[14] Safak M, Barrucco R, Darbinyan A, Okada Y, Nagashima K (2001). Interaction of JC virus agno protein with T antigen modulates transcription and replication of the viral genome in glial cells.. J Virol.

[15] Deshmane SL, Mukerjee R, Fan S, Del Valle L, Michiels C (2009). Activation of the oxidative stress pathway by HIV-1 Vpr leads to induction of hypoxia-inducible factor 1alpha expression.. J Biol Chem.

[16] Uleri E, Beltrami S, Gordon J, Dolei A, Sariyer IK Extinction of tumor antigen expression by SF2/ASF in JCV-transformed cells.. Genes Cancer.

[17] Gentilella A, Khalili K (2009). Autoregulation of co-chaperone BAG3 gene transcription.. J Cell Biochem.

[18] Kumar SH, Rangarajan A (2009). Simian virus 40 small T antigen activates AMPK and triggers autophagy to protect cancer cells from nutrient deprivation.. J Virol.

[19] Laderoute KR, Amin K, Calaoagan JM, Knapp M, Le T (2006). 5′-AMP-activated protein kinase (AMPK) is induced by low-oxygen and glucose deprivation conditions found in solid-tumor microenvironments.. Mol Cell Biol.

[20] Zhou G, Myers R, Li Y, Chen Y, Shen X (2001). Role of AMP-activated protein kinase in mechanism of metformin action.. J Clin Invest.

[21] Zhou J, Huang W, Tao R, Ibaragi S, Lan F (2009). Inactivation of AMPK alters gene expression and promotes growth of prostate cancer cells.. Oncogene.

[22] Zhuang Y, Miskimins WK (2008). Cell cycle arrest in Metformin treated breast cancer cells involves activation of AMPK, downregulation of cyclin D1, and requires p27Kip1 or p21Cip1.. J Mol Signal.

[23] Liu B, Fan Z, Edgerton SM, Deng XS, Alimova IN (2009). Metformin induces unique biological and molecular responses in triple negative breast cancer cells.. Cell Cycle.

[24] Wang W, Fan J, Yang X, Furer-Galban S, Lopez de Silanes I (2002). AMP-activated kinase regulates cytoplasmic HuR.. Mol Cell Biol.

[25] Orba Y, Suzuki T, Makino Y, Kubota K, Tanaka S Large T antigen promotes JC virus replication in G2-arrested cells by inducing ATM- and ATR-mediated G2 checkpoint signaling.. J Biol Chem.

[26] Spitz DR, Sim JE, Ridnour LA, Galoforo SS, Lee YJ (2000). Glucose deprivation-induced oxidative stress in human tumor cells. A fundamental defect in metabolism?. Ann N Y Acad Sci.

[27] Nath KA, Ngo EO, Hebbel RP, Croatt AJ, Zhou B (1995). alpha-Ketoacids scavenge H2O2 in vitro and in vivo and reduce menadione-induced DNA injury and cytotoxicity.. Am J Physiol.

[28] Christofk HR, Vander Heiden MG, Harris MH, Ramanathan A, Gerszten RE (2008). The M2 splice isoform of pyruvate kinase is important for cancer metabolism and tumour growth.. Nature.

[29] Gan DD, Khalili K (2004). Interaction between JCV large T-antigen and beta-catenin.. Oncogene.

[30] Khalili K, Del Valle L, Wang JY, Darbinian N, Lassak A (2003b). T-antigen of human polyomavirus JC cooperates withIGF-IR signaling system in cerebellar tumors of the childhood-medulloblastomas.. Anticancer Res.

[31] Izumi T, Io K, Matsui M, Shirakawa K, Shinohara M (2010). HIV-1 viral infectivity factor interacts with TP53 to induce G2 cell cycle arrest and positively regulate viral replication.. Proc Natl Acad Sci USA.

[32] Cheng P, Li Y, Yang L, Wen Y, Shi W (2009). Hepatitis B virus X protein (HBx) induces G2/M arrest and apoptosis through sustained activation of cyclin B1-CDK1 kinase.. Oncology Reports.

[33] Davy CE, Jackson DJ, Wang Q, Raj K, Masterson PJ (2002). Identification of a G(2) arrest domain in the E1 wedge E4 protein of human papillomavirus type 16.. J Virol.

[34] Kitagawa K, Nishino H, Iwashima A (1985). Analysis of hexose transport in untransformed and sarcoma virus-transformed mouse 3T3 cells by photoaffinity binding of cytochalasin B.. Biochim Biophys Acta.

[35] Bravard A, Beaumatin J, Luccioni C, Fritsch P, Lefrancois D (1992). Chromosomal, mitochondrial and metabolic alterations in SV40-transformed rabbit chondrocytes.. Carcinogenesis.

[36] Lachaise F, Martin G, Drougard C, Perl A, Vuillaume M (2001). Relationship between posttranslational modification of transaldolase and catalase deficiency in UV-sensitive repair-deficient xeroderma pigmentosum fibroblasts and SV40-transformed human cells.. Free Radic Biol Med.

[37] Kasai D, Adachi T, Deng L, Nagano-Fujii M, Sada K (2009). HCV replication suppresses cellular glucose uptake through down-regulation of cell surface expression of glucose transporters.. J Hepatol.

[38] Manel N, Kim FJ, Kinet S, Taylor N, Sitbon M (2003). The ubiquitous glucose transporter GLUT-1 is a receptor for HTLV.. Cell.

[39] Zwerschke W, Mazurek S, Massimi P, Banks L, Eigenbrodt E (1999). Modulation of type M2 pyruvate kinase activity by the human papillomavirus type 16 E7 oncoprotein.. Proc Natl Acad Sci USA.

[40] Maehama T, Patzelt A, Lengert M, Hutter KJ, Kanazawa K (1998). Selective down-regulation of human papillomavirus transcription by 2-deoxyglucose.. Int J Cancer.

[41] Jones JS, Risser R (1993). Cell fusion induced by the murine leukemia virus envelope glycoprotein.. J Virol.

[42] Sato H, Takimoto T, Tanaka S, Ogura H, Shiraishi K (1989). Cytopathic effects induced by Epstein-Barr virus replication in epithelial nasopharyngeal carcinoma hybrid cells.. J Virol.

[43] Dwarakanath BS, Singh D, Banerji AK, Sarin R, Venkataramana NK (2009). Clinical studies for improving radiotherapy with 2-deoxy-D-glucose: present status and future prospects.. J Cancer Res Ther.

[44] Hernlund E, Hjerpe E, Avall-Lundqvist E, Shoshan M (2009). Ovarian carcinoma cells with low levels of beta-F1-ATPase are sensitive to combined platinum and 2-deoxy-D-glucose treatment.. Mol Cancer Ther.

[45] Maschek G, Savaraj N, Priebe W, Braunschweiger P, Hamilton K (2004). 2-deoxy-D-glucose increases the efficacy of adriamycin and paclitaxel in human osteosarcoma and non-small cell lung cancers in vivo.. Cancer Res.

[46] Yamada M, Tomida A, Yun J, Cai B, Yoshikawa H (1999). Cellular sensitization to cisplatin and carboplatin with decreased removal of platinum-DNA adduct by glucose-regulated stress.. Cancer Chemother Pharmacol.

